# Human CART22.19 therapy in refractory pediatric B-ALL: insights from a named-patient cohort

**DOI:** 10.1136/jitc-2025-013901

**Published:** 2026-05-21

**Authors:** Anna-Sophia Mast, Peter Lang, Patrick Schlegel, Friso G Calkoen, Daniel Atar, Sophia Scheuermann, Sylvia Klein, Christiane Braun, Florian Schinle, Marina Schmidt, Luca Hensen, Martin Ebinger, Michaela Döring, Jürgen F Schäfer, Johannes Schulte, Wolfgang A Bethge, Claudia Lengerke, Bader Alahmari, Peirong Hu, Dina Schneider, Rimas J Orentas, Rupert Handgretinger, Christian Martin Seitz

**Affiliations:** 1 Department of General Pediatrics, Hematology and Oncology, University Hospital Tübingen, Tübingen, Germany; 2 Excellence cluster iFIT (EXC 2180) “Image-Guided and Functionally Instructed Tumor Therapies”, University Hospital Tübingen, Tübingen, Germany; 3 German Cancer Research Consortium (DKTK), Partner Site Tübingen, German Cancer Research Center, Heidelberg, Germany; 4 Department of Pediatric Hematology, Oncology and Stem Cell Transplantation, University of Regensburg, Regensburg, Germany; 5 Leibniz Institute for Immunotherapy, Regensburg, Germany; 6 Princess Máxima Center for Pediatric Oncology, Utrecht, Netherlands; 7 Hopp-Children’s Cancer Center Heidelberg (KiTZ), Heidelberg, Germany; 8 B310 Clinical Cooperation Unit Pediatric Oncology, German Cancer Research Center (DKFZ), Heidelberg, Germany; 9 Department of Pediatric Oncology, Hematology, and Immunology, Heidelberg University Hospital, Heidelberg, Germany; 10 Department of Hematology and Oncology, University Hospital Tübingen, Tübingen, Baden-Württemberg, Germany; 11 Department of Diagnostic and Interventional Radiology, University Hospital Tübingen, Tübingen, Germany; 12 King Abdullah International Medical Research Center, Riyadh, Riyadh Province, Saudi Arabia; 13 Department of Oncology, Ministry of National Guard Health Affairs, Riyadh, Riyadh Province, Saudi Arabia; 14 Lentigen Technology, Gaithersburg, Maryland, USA; 15 Research and Development Immunotherapy, Miltenyi Biotec BV & Co KG, Bergisch Gladbach, Germany

**Keywords:** Chimeric antigen receptor - CAR, Immunotherapy, Leukemia, T cell, Adoptive cell therapy - ACT

## Abstract

**Background:**

CD19-directed chimeric antigen receptor (CAR) T-cell therapies have transformed the treatment landscape for pediatric B-cell acute lymphoblastic leukemia (B-ALL), yet relapses driven by antigen escape remain a major limitation. Dual-targeting CAR approaches recognizing CD19 and CD22 have shown promising clinical activity, but sustained remissions are limited by insufficient CAR T-cell persistence.

**Methods:**

CAR22.19, a fully human tandem CD19/CD22 CAR, was developed and administered under a named-patient program to nine heavily pretreated pediatric patients with relapsed or refractory B-ALL. Treatment indications were CD19-negative blast population (n=5), relapse after CD19 CAR T (n=3) and/or restricted access to approved CAR T-cell products (n=3). Autologous and donor-derived CAR22.19 T-cells (CART22.19) were manufactured using a good manufacturing practice-compliant, semiautomated fresh-in-fresh-out process. Safety and efficacy were assessed through standardized clinical monitoring, measurable residual disease analysis, and CAR T-cell kinetics.

**Results:**

Preclinical validation demonstrated antigen-specific cytotoxicity and dual antigen activity. Clinically, CART22.19 were well tolerated, with no treatment-related deaths and no grade ≥3 neurotoxicity, while grade ≥3 cytokine release syndrome occurred in 38.5% (5/13) of infusions and resolved with standard interventions. An initial complete molecular remission was achieved in 78% (7/9) of patients, with a 12-month overall survival rate of 55.6% (95% CI, 20.4-80.5%). Complete remission in CD19⁻CD22⁺ disease underscores the functional contribution of the CD22-targeting domain, whereas all patients refractory to prior CD19 CAR T-cell therapy relapsed early despite retained CD19⁺CD22⁺ expression. Limited in vivo persistence may represent a contributing factor to treament failure. Notably, durable remission and sustained functional persistence of CART22.19 was achieved in one patient refractory to autologous CART22.19 following infusion of donor-derived CART22.19 after reduced-intensity conditioning (RIC) allogeneic hematopoietic stem cell transplantation (alloHSCT) in nonremission.

**Conclusions:**

CART22.19 therapy demonstrated a favorable safety profile and promising clinical activity in a high-risk pediatric population, with dual targeting enabling disease control in CD19-negative leukemia. Nonetheless, limited CAR T-cell persistence may represent an important obstacle to sustained remission. Our findings support further clinical development of CART22.19 and indicate that donor-derived CAR T-cells following RIC alloHSCT may represent a potential therapeutic strategy to enhance persistence and improve outcomes in heavily pretreated pediatric patients.

**WHAT IS ALREADY KNOWN ON THIS TOPIC**

**WHAT THIS STUDY ADDS**

- CART22.19 induced complete molecular remissions in 78% of patients, including those with CD19-negative disease. However, patients who had relapsed after prior CD19 CAR T-cell therapies exhibited poor outcomes, and relapses after CART22.19 appeared to be associated with limited in vivo CAR T-cell persistence. Durable remission was achieved in one patient following donor-derived CART22.19 therapy after reduced-intensity conditioning (RIC) allogeneic hematopoietic stem cell transplantation (alloHSCT), suggesting a potential strategy to enhance persistence and improve outcomes in heavily pretreated pediatric B-ALL.

**HOW THIS STUDY MIGHT AFFECT RESEARCH, PRACTICE OR POLICY**

- These findings support further trials of dual-targeting CARs, including CART22.19, as well as strategies to enhance in vivo CAR T-cell persistence. In addition, donor-derived CAR T-cell therapy following RIC alloHSCT may warrant further investigation in refractory disease.

## Introduction

 The advent of CD19-targeted chimeric antigen receptor (CAR) T-cells (CAR19) has revolutionized the therapy of pediatric acute lymphoblastic leukemia (ALL).^1–5^ While initial complete remission (CR) rates are high (70–90%), the interpretation of long-term event-free survival (EFS) remains challenging due to heterogeneity in post-remission treatment strategies. Specifically, a variable proportion of patients (13–88%) undergo consolidative allogeneic hematopoietic stem cell transplantation (alloHSCT), which complicates outcome comparisons across studies.^2 5 6^

Relapse following CAR19 therapy generally manifests in two distinct patterns: CD19-negative relapse, often attributed to effective elimination of CD19-positive cells by potent CAR T-cell products, or CD19-positive relapse, which is associated with limited CAR T-cell functionality or persistence.^3 4 7–14^ Antigen escape, characterized by the loss or modulation of the targeted surface antigen, poses a significant challenge in both CD19-targeted and CD22-targeted CAR T-cell therapies.^3 15 16^

To counteract antigen-negative escape, dual-targeting CAR T-cell approaches have been developed, particularly by combining CD19 and CD22. Dual targeting can be achieved through several strategies^1^: coadministration of two monospecific CAR T-cell products,^2^ cotransduction of T-cells with two separate vectors,^3^ adapter CAR platforms that enable modular antigen recognition,^4^ the use of bicistronic vectors encoding both CARs, or^5^ tandem CAR constructs incorporating two antigen-binding domains in a single receptor.^17–24^ While coadministration and cotransduction strategies enable rapid clinical translation using validated components, they result in heterogeneous cell populations and increase manufacturing complexity. In contrast, bicistronic and tandem constructs yield uniform dual-targeting CAR T-cell populations, ensuring all transduced cells recognize both antigens and offering potential manufacturing and economic advantages.

Despite promising safety data, dual CD19/CD22-directed CAR T-cell therapies have yet to demonstrate consistent superiority over monospecific CAR19 with respect to long-term outcomes. Reported 1-year EFS rates range widely from 32% to 83%.^17–21 25^ Limited CAR T-cell persistence is frequently cited as a major contributor to relapse, although the mechanisms underlying CD19-positive relapse remain incompletely understood.^17–19 21 26^ Contributing factors may include prior cytotoxic treatments, T-cell exhaustion, or immune-mediated rejection.

We report the development and clinical application of CAR22.19, a fully human tandem CAR targeting CD19 and CD22. Within a named-patient program, nine pediatric patients with relapsed or refractory B-cell ALL (B-ALL) received CART22.19. We provide an overview of clinical outcomes in the cohort, followed by an in-depth case description of a patient who achieved long-term remission after receiving donor-derived CART22.19 following reduced-intensity conditioning (RIC) alloHSCT in non-remission.

## Methods

### Design of the CAR22.19 Construct

**Figure 1:**
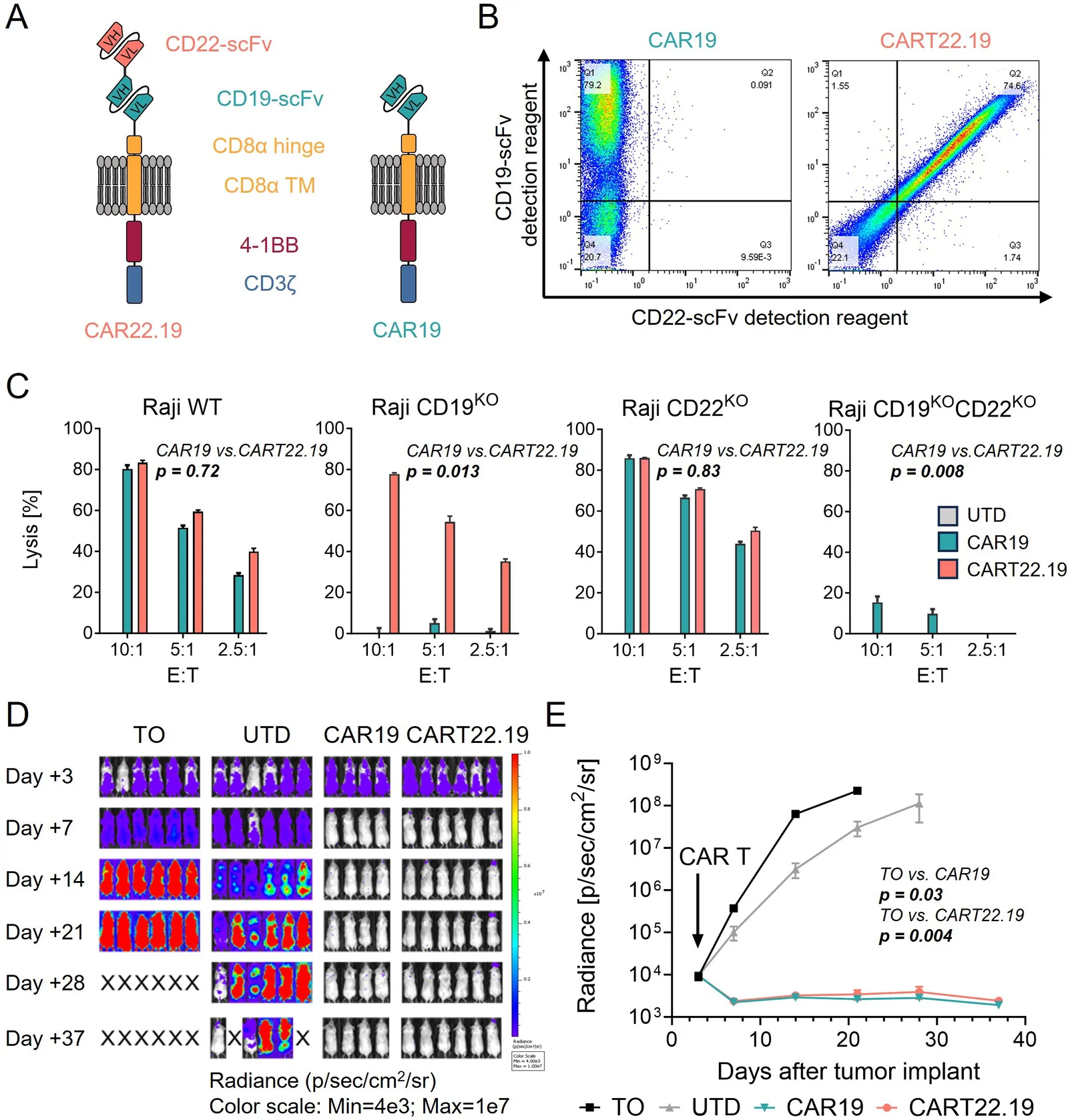
Design and preclinical evaluation of CAR22.19. (**A**) Schematic of the fully human CAR22.19 compared with a conventional CAR19: an anti-CD22 scFv fused in-frame to an anti-CD19 scFv, linked to a CD8α hinge and transmembrane region and incorporating a 4-1BB costimulatory and CD3ζ signaling domain. (**B**) Surface expression of CAR constructs was assessed by flow cytometry using binder-specific detection reagents for the CD19-targeting and CD22-targeting scFv domains. (**C**) CART22.19, CAR19, or UTD were co-cultured with target cells at indicated E:T ratios for 16 hours. Target cell lysis was quantified by luminometry to determine the percentage of cell lysis. Targets were Raji WT (CD19^+^CD22^+^), Raji CD19^KO^ (CD19⁻CD22^+^), Raji CD22^KO^ (CD19^+^CD22⁻), and Raji CD19^KO^CD22^KO^ (CD19^−^CD22^−^). Values represent the mean±SD of three technical replicates. Statistical significance was assessed using unpaired t-tests. (**D–E**) In vivo evaluation of CART22.19 in a NALM-6 xenograft model: NSG mice received 1×10^6^ firefly luciferase–expressing NALM-6 cells intravenously on day 0, followed by 1×10^7^ effector cells (CART22.19, CAR19, or UTD) on day 3. (**D**) Representative BLI images at indicated time points. Color scale represents radiance from 4×10^3^ to 1×10^7^ p/sec/cm²/sr. (**E**) Quantification of BLI radiance over time. Data represent mean±SEM; n=4–6 mice per group. Statistical significance was assessed using a mixed-effects model with Geisser-Greenhouse correction. BLI, bioluminescence imaging; CAR, chimeric antigen receptor; CAR19, CD19-directed CAR; CAR22.19, fully human tandem CAR with anti-CD22 and anti-CD19 scFvs; CART22.19, CAR22.19 T-cells; E:T, effector-to-target; KO, knockout; NSG, NOD scid gamma; p, photons; scFv, single-chain variable fragment; sec, second; sr, steradian; TM, transmembrane domain; TO, tumor only control; t-test, unpaired Student’s t-test; UTD, untransduced activated T-cells; VH, variable heavy; VL, variable light; WT, wild type.

The fully human CAR22.19 construct consists of a tandem targeting domain, comprising an anti-CD22 single-chain variable fragment (scFv; clone 16p17) fused in-frame to an anti-CD19 scFv (clone M19217-1). Within each scFv, the variable heavy and variable light domains are connected by a flexible linker consisting of three repeats of the glycine-serine motif (G4S)3 (15 amino acids; 45 base pairs). The two scFvs are joined by an inter-scFv linker composed of five repeats of the G4S motif (G4S)₅ (25 amino acids; 75 base pairs). The tandem scFv is linked to a CD8α hinge and transmembrane region, followed by a 4–1BB costimulatory domain and a CD3ζ signaling domain. A schematic representation of the CAR22.19 structure is provided in figure 1A.

### Clinical scale manufacturing of CART22.19

The investigational product CART22.19 was manufactured from autologous or allogeneic T-cells obtained by leukapheresis from patients or their HSCT donors. Cells were processed freshly without cryopreservation and modified genetically to express the CAR22.19 construct. Manufacturing was performed under cleanroom conditions at the good manufacturing practice (GMP)-certified facility of the University Hospital Tübingen (UKT), following a standardized 12-day T-cell culture and transduction protocol using the CliniMACS Prodigy platform (Miltenyi Biotec), as described previously.^27^ We employed a GMP-grade, third-generation lentiviral vector encoding the CAR22.19 construct under the control of the human elongation factor 1α promoter. Lentivirus was kindly provided by Miltenyi Biotec.

All CAR T-cell products underwent comprehensive quality control testing, including assessments of sterility, cell viability, transduction efficiency, and other predefined release criteria, and were administered freshly without cryopreservation. Summary data from in-process monitoring and final product testing are provided in online supplemental table S1. Additional information on lentiviral vector production and preclinical validation (in vitro and in vivo) is included in the online supplemental methods.

### Patient cohort

This report summarizes outcomes from individually authorized named-patient treatments. Informed consent was obtained from all patients or their legal guardians following detailed counseling by the treating physicians.

All patients had relapsed or refractory disease with no remaining standard treatment options and met at least one of the following criteria: relapse following prior therapy with an approved CAR19 product (n=3), presence of CD19-negative leukemic blast populations (n=5), or lack of access to a licensed CAR19 therapy (n=3). Treatment with CART22.19 was recommended by the interdisciplinary Cell Therapy Board at the UKT.

Autologous CART22.19 infusions were administered to patients without a history of HSCT or lacking access to the original donor (n=7), whereas donor-derived allogeneic infusions were given to patients with prior alloHSCT and an available original donor (n=3). Among patients with prior alloHSCT who subsequently received autologous products with T-cells isolated post-transplant, 3/4 exhibited mixed chimerism at the time of apheresis due to disease relapse. Upon relapse, two patients received two additional CART22.19 infusions from cryopreserved products as approved by the Cell Therapy Board, resulting in a total of thirteen infusions across nine patients.

### CAR administration

Lymphodepleting chemotherapy was initiated at day −5 or −4 prior to CAR T-cell infusion and consisted of fludarabine at 25 mg/m² body surface area (BSA) once daily for three consecutive days, combined with a single dose of cyclophosphamide at 900mg/m² BSA. In one patient (P5), lymphodepletion was initiated earlier (day −12) due to the presence of a BCR-ABL1 fusion with a p.T315I mutation, and ponatinib was administered from day −7 to day −2 in addition to lymphodepleting chemotherapy. In select cases, the regimen was adjusted at the discretion of senior physicians based on individual risk factors and anticipated toxicity. CART22.19 was infused on day 0 at a median dose of 3× 10⁶ cells/kg body weight (BW) (range, 0.3–6.0 × 10⁶ cells/kg BW), with dose modifications applied in selected cases due to safety considerations (eg, allogeneic donor-derived products or prior severe cytokine release syndrome (CRS)) or, conversely, dose escalation in the setting of good tolerability and high therapeutic urgency.

### Therapy response and safety assessment

Treatment response was evaluated by bone marrow minimal residual disease (MRD) analysis using real-time quantitative PCR or multiparameter flow cytometry, and by imaging studies in cases with extramedullary involvement. CR was defined as MRD negativity in the bone marrow and the absence of extramedullary disease. Treatment-related toxicities, including CRS and immune effector cell-associated neurotoxicity syndrome (ICANS), were graded according to the criteria established by the American Society for Transplantation and Cellular Therapy (ASTCT) and the Common Terminology Criteria for Adverse Events (CTCAE), V.5.0.^28 29^ Laboratory parameters were monitored routinely during treatment and follow-up to assess toxicity and treatment response. Detailed methodologies are described in the online supplemental methods.

### Outcomes

All patients were monitored up to the data cut-off of February 28, 2026. EFS was defined as the time from CART22.19 infusion to the first documented event: MRD reappearance, clinical relapse, or death from any cause. In patients who received more than one CART22.19 infusion, EFS was calculated from the date of the latest administration. Patients undergoing subsequent HSCT were not censored. Overall survival (OS) was measured from the date of the first CART22.19 infusion to death from any cause. Duration of remission was defined as the time from achievement of complete molecular remission to relapse. For patients who received more than one CART22.19 infusion, remission duration was calculated from the latest infusion.

### Data analysis

Objective response rates as well as EFS and OS rates were reported with two-sided 95% CIs, calculated using the Clopper-Pearson method. Survival curves were estimated using the Kaplan-Meier method. All subgroup analyses were conducted post hoc and reported descriptively due to the small sample size and non-interventional nature of the dataset. No formal hypothesis testing was performed. Statistical significance was defined as p <0.05. Analyses were performed using GraphPad Prism (V.9.4.1) and flow cytometry data were analyzed using FlowJo (V.10.8).

## Results

### Preclinical Evaluation of CART22.19

To overcome antigen escape through CD19 loss and reduce immunogenicity associated with murine-derived scFvs, we developed a fully human tandem CAR22.19 (figure 1A). Transduction of T-cells with the CAR22.19 construct resulted in stable surface expression, with both binding domains assessable for target binding (figure 1B). Functional characterization demonstrated antigen-specific cytotoxicity and cytokine secretion (interleukin-2, interferon-gamma) in response to CD19^+^CD22^+^ Raji cell coculture as well as CD19 or CD22 single-knockout variants, with no activity against double-knockout cells lacking both targets (figure 1C; online supplemental figure S1A,B). In contrast to monospecific CAR19 T-cells, CART22.19 were activated by either antigen, CD19 or CD22.

In vivo, CART22.19 induced rapid and sustained tumor clearance, comparable to CAR19 T-cells (figure 1D and E) and persisted with a predominant central memory phenotype (online supplemental figure S1C,D). These results strongly supported clinical translation of CART22.19 and prompted the initiation of a named-patient program.

### Patient characteristics

Based on the promising preclinical results, nine pediatric patients with relapsed or refractory B-ALL were treated with CART22.19 within a named-patient program between November 2019 and June 2024. All patients had exhausted standard therapeutic options and were ineligible for approved commercial CAR T-cell therapies. Five patients presented with CD19-negative leukemic blast populations, three had relapsed after previous CAR19 therapy, and in three cases, regulatory restrictions prevented access to commercial products.

CART22.19 were manufactured using a semi-automated process on the CliniMACS Prodigy platform, achieving a median transduction efficiency of 53% (range, 48.3–70.8%) (online supplemental table S1). Thirteen infusions were administered across the nine patients, with two individuals receiving three separate infusions due to persistent or recurrent disease. Seven patients received autologous CAR T-cell products, while three were treated with donor-derived CART22.19. The most frequently administered dose was 3×10⁶ CART22.19/kg BW, used in eight of the thirteen infusions, with an overall dosing range of 0.3×10⁶ to 6×10⁶ CART22.19/kg BW.

Patient characteristics are summarized in table 1, with detailed information on individual patients provided in online supplemental table S2. The median age at initial diagnosis was 10.2 years (range, 0.2–16.6 years). All patients were heavily pretreated, with a median of four prior therapy lines. Seven patients had received prior anti-CD19 and/or anti-CD22 therapies, three had relapsed following approved CAR19 T-cell products, and six had experienced relapse after alloHSCT. The number of prior relapses ranged from 0 to 6, with a median interval of 51.5 months from initial diagnosis to CART22.19 infusion (range, 8–72 months). The disease status prior to lymphodepletion was heterogeneous: six infusions were given for MRD-level disease, five for overt hematologic relapse, one for progressive extramedullary disease with MRD-level disease (P1), and one for isolated central nervous system (CNS) involvement without bone marrow infiltration (P5).

**Table 1:** Clinical characteristics and outcomes of pediatric patients treated with CART22.19

Patient ID	Indication for CART22.19	CART22.19 source	Dose level (x10^6^/kg BW)	Initial response to CART22.19	Relapse after CART22.19 (yes=1/no=0)	CRS (grade)	ICANS (grade)
P1	CD19 neg blast pop	2× Auto/1× MUD	3/6/3	CR/ PD/CR	0/0/0	3/0/3	0/0/0
P2	CD19 neg blast pop	Auto	3	CR	0	1	0
P3	Relapse after CAR19, CD19 neg blast pop	Auto	3	PD	0	0	0
P4	Relapse after CAR19	Auto	3	CR	1	2	1
P5	No access to CAR19	MSD	1	PD	0	2	0
P6	Relapse after CAR19, CD19 neg blast pop	MMFD	1	CR	1	3	0
P7	No access to CAR19	Auto	3	CR	1	1	0
P8	No access to CAR19	3× Auto	3/0.3/5	CR/PD/CR	0/0/0	4/2/0	0/0/0
P9	CD19 neg blast pop	Auto	3	CR	0	4	1

Auto, autologous; BW, body weight; CAR, chimeric antigen receptor; CAR19, CD19 directed CAR T-cells; CR, complete remission; CRS, cytokine release syndrome; ICANS, immune effector cell-associated neurotoxicity syndrome; MMFD, mismatched family donor; MSD, matched sibling donor; MUD, matched unrelated donor; neg, negative; PD, persistent/progressive disease; pop, population.

Collectively, this cohort represents a highly heterogeneous and heavily pretreated group of non-selected pediatric patients with no remaining curative treatment options. The named-patient use of CART22.19 in this context highlights a pressing unmet clinical need.

### Adverse events

The safety profile of CART22.19 therapy was evaluated across all thirteen infusions (table 2). Treatment-emergent toxicities within the first 28 days post-infusion were graded according to CTCAE and ASTCT criteria.^28 29^ Adverse events (AEs) of any grade occurred following 11 of 13 infusions, most frequently fever (84.6%), alanine aminotransferase elevation (69.2%), and aspartate aminotransferase elevation (61.5%). Grade ≥3 AEs were observed after 10 infusions, most commonly neutropenia (76.9%), anemia (69.2%), and hypotension (38.5%). All grade 3 cytopenia resolved within 2 months. Grade ≥3 CRS occurred in five cases (38.5%) and resolved within 2 weeks following standard management per institutional guidelines. ICANS was observed in two patients, both presenting with mild symptoms (headache, dizziness). One patient (P5) with pre-existing CNS leukemia experienced recurrent seizures and somnolence before and after CART22.19 infusion. In the absence of CRS, elevated cytokines, or other ICANS features, these events were attributed to disease progression. CRS and ICANS were managed with tocilizumab (77%), dexamethasone (46%), and/or anakinra (15%), with complete resolution in all cases. Additional isolated events included sepsis, pancreatitis, and transient adrenal insufficiency. One patient (P8) developed severe catheter-related sepsis with septic shock, which resolved under appropriate therapy. No cases of treatment-related death or hemophagocytic lymphohistiocytosis were reported, and no infection-related mortality occurred. Overall, CART22.19 therapy demonstrated a manageable safety profile, with AEs effectively controlled using established supportive care protocols, including regular immunoglobulin monitoring and intravenous immunoglobulin substitutions in the setting of B-cell aplasia.

**Table 2:** Adverse events

	Any grade	Grade ≥3
**All adverse events**	13 (100%)	13 (100%)
Neutropenia	2 (15.4%)	10 (76.9%)
Anemia	3 (23.1%)	9 (69.2%)
Fever	11 (84.6%)	0 (0%)
ALT increase	9 (69.2%)	0 (0%)
AST increase	8 (61.5%)	0 (0%)
Hypoalbuminemia	7 (53.8%)	0 (0%)
LDH increase	6 (46.1%)	0 (0%)
Hypotension	0 (0%)	5 (38.5%)
Myalgia	0 (0%)	3 (23.1%)
Hypoxia	2 (15.4%)	1 (7.7%)
Necessity of parenteral nutrition	3 (23.1%)	0 (0%)
Hypofibrinogenemia	2 (15.4%)	0 (0%)
Creatinine increase	2 (15.4%)	0 (0%)
Delir	1 (7.7%)	2 (15.4%)
Mucositis	0 (0%)	2 (15.4%)
Alkaline phosphatase increase	2 (15.4%)	0 (0%)
Diarrhea	2 (15.4%)	0 (0%)
Headache	2 (15.4%)	0 (0%)
Neuropathic pain	2 (15.4%)	0 (0%)
Seizure	0 (0%)	1 (7.7%)
Sepsis	0 (0%)	1 (7.7%)
Pancreatitis	1 (7.7%)	0 (0%)
Transient adrenal insufficiency	1 (7.7%)	0 (0%)
**CRS**	6 (46.1%)	5 (38.5%)
**ICANS**	2 (15.4%)	0 (0%)
**Death**	0 (0%)	0 (0%)

AEs were graded per CTCAE V.5.0; CRS and ICANS per ASTCT criteria.

AEs, adverse events; ALT, alanine aminotransferase; AST, aspartate aminotransferase; ASTCT, American Society for Transplantation and Cellular Therapy; CRS, cytokine release syndrome; CTCAE, Common Terminology Criteria for Adverse Events; ICANS, immune effector cell-associated neurotoxicity syndrome; LDH, lactate dehydrogenase.

### Clinical outcomes

**Figure 2:**
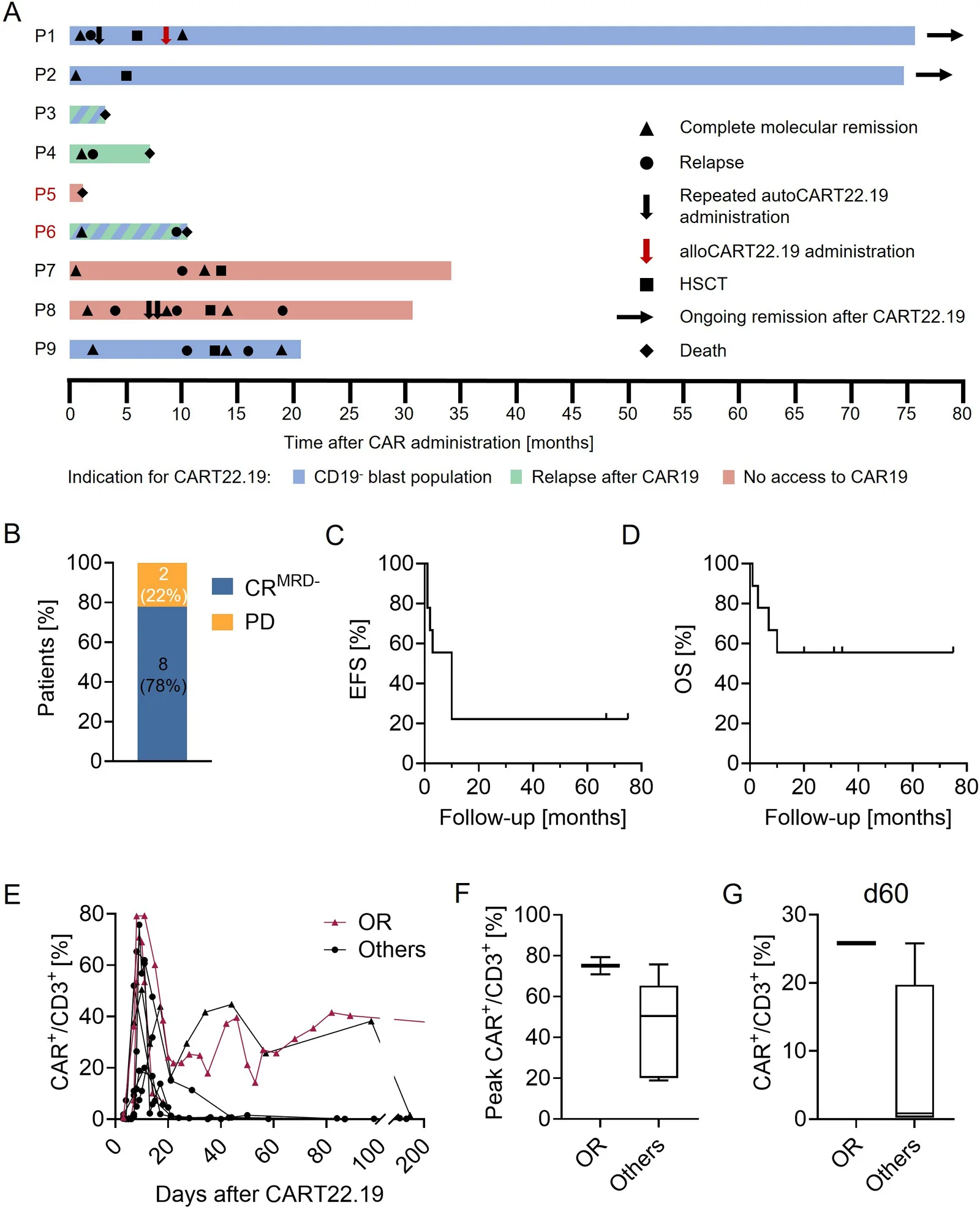
Clinical outcomes following CART22.19 therapy. (**A**) Swimmer plot illustrating the postinfusion clinical course for each patient. The indication for CART22.19 therapy is color-coded: blue indicates a CD19-negative blast population, green relapse after prior CAR19 therapy, and red cases where patients had no access to CAR19 therapy. Patient numbers in red refer to allogeneic CART22.19 recipients; black numbers indicate autologous CART22.19 recipients. (**B**) Initial response rates following CART22.19 administration, categorized as complete molecular remission or persistent/progressive disease. For patients who received multiple CART22.19 infusions, the response is assigned to the latest infusion. (**C**) EFS after CART22.19 administration within the observation period; events were defined as molecular or clinical relapse or death. (**D**) OS of patients following CART22.19 therapy. (**E**) Proportional dynamics of CAR^+^ cells among the CD3^+^ T-cells after CART22.19 administration; patients with ongoing remission are shown in red, others in black. (**F**) Peak percentage of CAR^+^ cells among the CD3^+^ T-cells, comparing patients with ongoing remission to others. (**G**) Frequency of CAR^+^ cells within CD3^+^ T-cells on day 60 postinfusion, comparing patients with ongoing remission to others. EFS and OS (**C**, **D**) were estimated using the Kaplan-Meier method; comparison in panel **F** showed no significant differences (p=0.11; Mann-Whitney U test). CAR, chimeric antigen receptor; CAR19, CD19-directed CAR; CART22.19, fully human tandem CAR T-cells with anti-CD22 and anti-CD19 scFvs; CR, complete remission; d60, day 60 postinfusion; EFS, event-free survival; HSCT, hematopoietic stem cell transplantation; MRD−, minimal residual disease negative; OR, ongoing remission; OS, overall survival; PD, persistent/progressive disease.

The clinical courses of the nine pediatric patients treated with CART22.19 are summarized in figure 2A, illustrating treatment responses, remission durability, and overall outcomes in this high-risk, heavily pretreated cohort. As of the data cut-off, median follow-up was 34.2 months (range, 20.4–75.4). At the initial response assessment (30–60 days postinfusion), seven of nine patients had achieved complete molecular remission (figure 2B). Two patients (P1, P2) remain in ongoing remission. Notably, P1 achieved CR after receiving allogeneic CART22.19 following relapse and non-response to two prior autologous infusions and alloHSCT in non-remission. P2 achieved CR after a single CART22.19 infusion and subsequently underwent consolidative HSCT. Six patients achieved MRD-negative CR but relapsed subsequently. Among responders, the median remission duration was 8.7 months (range: 1.3–74.3 months), with a median time to relapse of 8.5 months (range: 1.3–9.2 months). Three of these patients (P7, P8, P9) underwent alloHSCT after relapse, and P7 and P9 are in remission at last follow-up. Patient P8 achieved MRD-negative CR following two autologous CART22.19 infusions but experienced two relapses, indicating only transient disease control. Two patients (P3 and P5) did not achieve CR following CART22.19 therapy and succumbed to disease progression shortly after treatment. Notably, all patients with prior refractoriness to CAR19 T-cell therapy relapsed within 10 months of CART22.19 infusion. At the time of data cut-off, five of the nine patients (55.6%) were alive.

Kaplan-Meier analysis showed EFS rates of 55.6% at 6 months (95% CI 20.4% to 80.5%) and 22.2% at both 12 and 24 months (95% CI 3.4% to 51.3%), with events defined as molecular or clinical relapse or death (figure 2C). OS rates were 77.8% at 6 months (95% CI 36.5% to 94%) and 55.6% at both 12 and 24 months (95% CI 20.4% to 80.5%) (figure 2D). Median OS was not reached. Taken together, these results demonstrate that tandem CART22.19 therapy can induce complete and durable remissions in a subset of heavily pretreated pediatric patients with B-ALL.

### Immunophenotypic characteristics and CART22.19 Kinetics

Immunophenotypic analysis of leukemic blasts prior to CART22.19 infusion revealed heterogeneous target antigen expression. CD19 expression was positive in three patients (P4, P7, P8), heterogeneous in three (P1, P2, P6), and absent in two patients (P3 and P9) with isolated CD22^+^CD19^−^ disease (online supplemental figure S2). CD22 expression was positive in six patients and heterogeneous in one (P1). No antigen expression data prior to treatment were available for P5, and CD22 expression was not assessed in P6.

Among the six patients who relapsed, one developed CD19^−^CD22^+^ disease after initially coexpressing both targets, while two relapsed with persistent CD19^+^CD22^+^ expression. Antigen expression data at relapse were not available for the remaining three patients. Notably, P9 achieved an initial CR despite complete absence of CD19 expression, highlighting the therapeutic contribution of the CD22-targeting component of the CAR22.19 construct.

CART22.19 expansion in peripheral blood was observed following 11 of 13 infusions, with peak levels occurring between days 7 and 15 (median, day 9). Peak frequencies of CART22.19 among peripheral T-cells ranged from 18.9% to 79.3%. One patient with ongoing response (P1) maintained CART22.19 frequencies of over 40% at day 90 postinfusion. No data beyond day 20 was available for the second patient in ongoing remission (P2; figure 2E). In contrast, patients who did not achieve long-term remission generally exhibited low or rapidly declining CART22.19 frequencies. Notably, P9 demonstrated a marked decline in CART22.19 frequency by day 180 postinfusion, followed by disease relapse a few months later. The mean peak CART22.19 frequency was 75.1% in patients with ongoing responses compared to 44.9% in others (figure 2F). By day 60, P1 exhibited a CART22.19 frequency of 25.8%, compared to a mean expression of 6.9% in patients who subsequently relapsed (figure 2G). Although these findings suggest a potential association between CART22.19 persistence and sustained clinical response, statistical significance was not reached due to limited sample size.

In summary, initial CR in a patient with CD19-negative disease highlights the therapeutic relevance of CD22 targeting, while the association between declining CAR T-cell levels and relapse implicates limited CART22.19 persistence as a key mechanism of treatment failure.

### Exploratory analysis of factors associated with ongoing remission

An exploratory subgroup analysis was performed to identify clinical factors potentially associated with ongoing remission following CART22.19 therapy (online supplemental figure S3). Among the nine pediatric patients treated, two (22%) achieved ongoing remission at a median follow-up of 34.2 months.

All continued responders were from the subgroup with CD19-negative leukemic subpopulations (40% (95% CI 5.3% to 85.3%)), whereas no patient treated for relapse after CAR19 T-cell therapy or for lack of access to commercial products achieved an ongoing response (0% in both groups (95% CI 0% to 70.8%)). Sustained remission was also more frequent in patients with fewer than three prior relapses (50% (95% CI 6.8% to 93.2%)) and in those treated within 50 months of initial diagnosis (50% (95% CI 6.8% to 93.2%)) compared with later treatment (0% (95% CI 0% to 60.2%)).

Other baseline characteristics, including age, immunophenotype (common vs pre-B ALL), and number of prior treatment lines, showed no apparent association with treatment outcome. Given the limited sample size, none of these trends showed statistical significance and should be interpreted with caution.

Nevertheless, these findings suggest that CD19-negative leukemic subpopulations, shorter intervals from initial diagnosis to CART22.19 treatment, and fewer prior relapses may be associated with improved long-term outcomes. These observations warrant confirmation in larger patient cohorts.

### Long-term Remission after Combined RIC AlloHSCT and Donor-derived CART22.19

**Figure 3:**
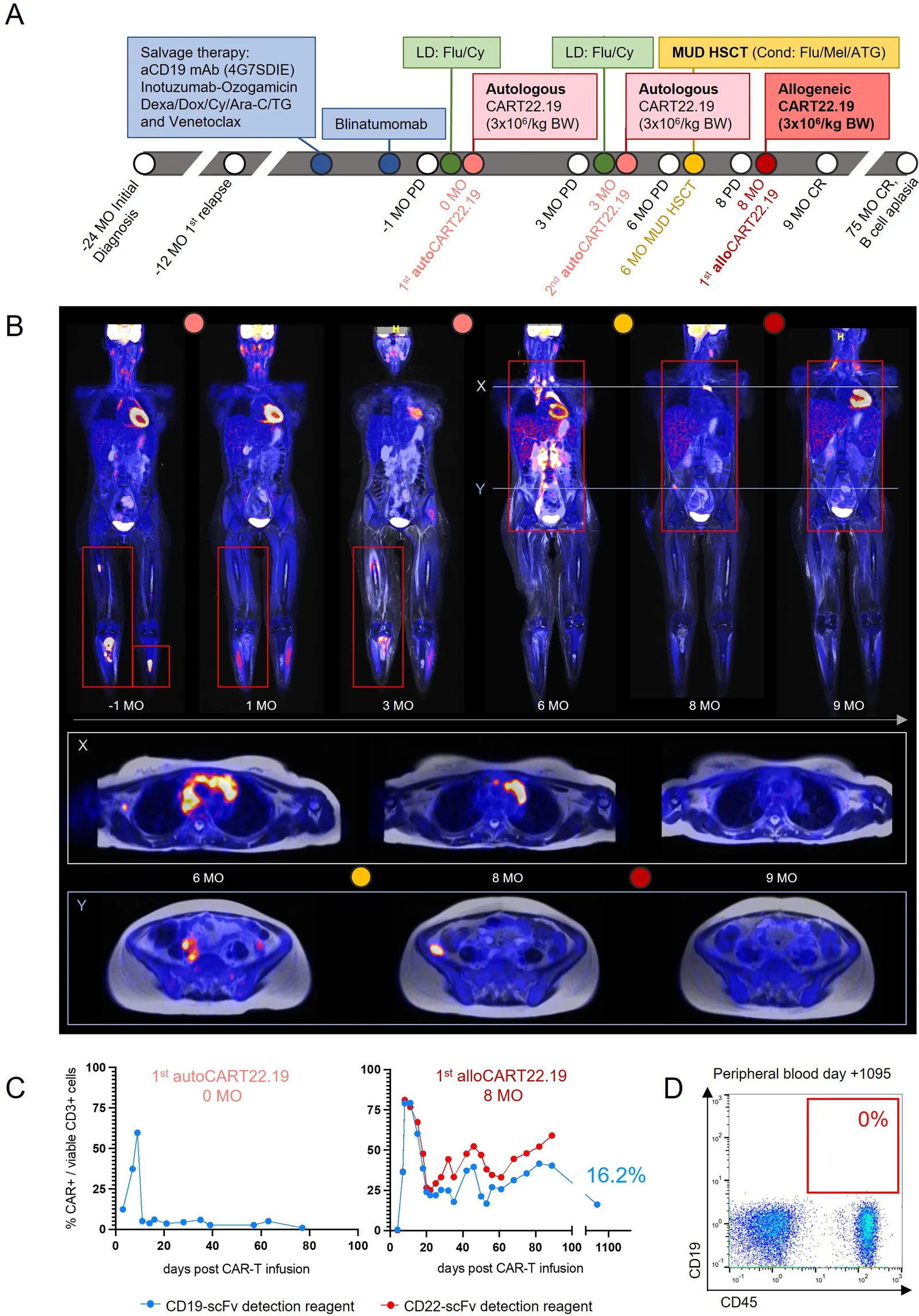
Allogeneic CART22.19 after RIC alloHSCT: clinical outcomes and CAR T kinetics. (**A**) Schematic overview of the clinical course of patient P1. Time points indicate months since first CART22.19 administration. (**B**) Whole-body ^18^F-FDG PET-MRI at selected time points, referenced to months since first CART22.19 administration. The upper panel shows coronal images with red boxes marking sites of disease manifestation. X and Y indicate the axial slices shown in the middle and lower panels. Colored dots correspond to the timing of therapeutic interventions shown in panel A. (**C**) Monitoring of CART22.19 dynamics in peripheral blood. Flow cytometric analysis of PBMCs at the indicated time points post-infusion, with CAR^+^ cells shown as a percentage of CD3^+^ T-cells using CD19-specific (blue) and CD22-scFv-specific reagents (red). The left panel depicts expansion after the first autologous CART22.19 infusion, and the right panel after the allogeneic CART22.19 infusion. (**D**) Flow cytometric analysis of peripheral blood on day+1095 postinfusion, demonstrating persistent B-cell aplasia. CD19 and CD45 are shown. alloHSCT, allogeneic hematopoietic stem cell transplantation; Ara-C, cytarabine; ATG, antithymocyte globulin; BW, body weight; CAR, chimeric antigen receptor; CART22.19, fully human tandem CAR T-cells with anti-CD22 and anti-CD19 scFvs; CR, complete remission; Cy, cyclophosphamide; Dexa, dexamethasone; Dox, doxorubicin; ^18^F-FDG, fluorine-18 fluorodeoxyglucose; Flu, fludarabine; LD, lymphodepletion; mAb, monoclonal antibody; Mel, melphalan; MO, months; MUD, matched unrelated donor; PD, progressive disease; PET-MRI, positron emission tomography-MRI; RIC, reduced-intensity conditioning; scFv, single-chain variable fragment; TG, thioguanine.

A detailed case presentation of P1 is provided in figure 3A, illustrating an innovative strategy to overcome resistance to CART22.19. When enrolled in the named-patient program, P1 presented with refractory pre-B ALL, including extensive skeletal involvement detected by ¹⁸F-FDG-PET-MRI (fluorine-18 fluorodeoxyglucose positron emission tomography-MRI; figure 3B; online supplemental figure S4A) and CD19-negative blast populations, after five prior lines of therapy including inotuzumab ozogamicin and blinatumomab. Following standard lymphodepletion with fludarabine and cyclophosphamide, fresh autologous CART22.19 (autoCART22.19) were infused at a dose of 3×10⁶ CAR^+^ cells/kg BW. Grade 3 CRS developed and was effectively managed with tocilizumab and transient vasopressor support, resolving within 2 weeks. No neurotoxicity was observed. The patient achieved radiologic and molecular CR with a peak CAR T-cell expansion of 60% of viable T-cells (996 CAR T-cells/μL) on day 9 and detectable levels persisting through day +77 (figure 3C; online supplemental figure S4C). Relapse occurred 3 months after autoCART22.19. A second infusion of cryopreserved autoCART22.19 did not show signs of clinical efficacy, with neither CRS nor measurable CART22.19 expansion and no objective response. Re-staging revealed progressive disease with bulky lymphadenopathy in multiple regions (figure 3B). Despite active disease, alloHSCT was performed using a TCRαβ/CD19-depleted graft from a 9/10 HLA-matched unrelated donor following bridging chemotherapy and RIC, without post-transplant immunosuppression.

Post-transplant evaluation demonstrated persistent extramedullary disease and rising MRD (2×10^−^⁴). On day +72 post HSCT, the patient received fresh donor-derived allogeneic CART22.19 (alloCART22.19) at a dose of 3×10⁶ CAR^+^ cells/kg BW, without additional lymphodepletion. Grade 3 CRS occurred but was effectively controlled and no ICANS or graft-versus-host-disease (GvHD) exacerbation was observed (online supplemental figure S4B). By day +17 post CART22.19, complete molecular and radiologic remission was achieved (figure 3B). Throughout the course of alloHSCT and alloCART22.19 therapy, no severe or chronic viral or fungal infections were reported.

Immune monitoring demonstrated distinct CAR T-cell expansion kinetics across the three infusions. Maximum CAR expansion of the alloCART22.19 product was observed on day +15 at 67% (1,110 CAR T-cells/μL), with persistence at 851 cells/µL through day +84 (figure 3C and online supplemental figure S2C). 4.5 years after infusion, alloCART22.19 continue to comprise 16% of circulating T-cells and retain functional activity, as evidenced by sustained B-cell aplasia (figure 3D). At the time of data cut-off, the patient remains in ongoing CR.

This case suggests that the combination of RIC alloHSCT and donor-derived CART22.19 therapy may be associated with long-term disease control in refractory leukemia and warrants further investigation.

## Discussion

This report presents the preclinical validation and initial clinical experience with CART22.19, a fully human tandem CAR T-cell therapy targeting CD19 and CD22, in a cohort of heavily pretreated pediatric patients with relapsed or refractory B-ALL. In vitro and in vivo analyses confirmed potent, antigen-specific cytotoxicity and dual-target activation. Clinically, CART22.19 was administered in a named-patient program to nine pediatric patients lacking standard curative treatment options.

The safety profile of CART22.19 was manageable and consistent with other published CAR T-cell studies in pediatric B-ALL, including the pivotal ELIANA trial evaluating tisagenlecleucel, which ultimately led to it’s regulatory approval.^6 17–21 25^ Based on ASTCT criteria, grade ≥3 CRS occurred in 38.5% of infusions, a rate that remained consistent when retrospectively applying the University of Pennsylvania grading scale used in ELIANA.^30^ While ELIANA reported grade 3–4 CRS in 25% and grade ≥3 neurotoxicity in 13.3% of patients, no severe ICANS occurred in our cohort.^6^ Notably, no treatment-related deaths were observed, in contrast to a study by Wang *et al*, which reported three fatalities among 225 patients receiving coadministered monospecific CD19-directed and CD22-directed CAR T-cells.^17^ These findings suggest a favorable safety profile of CART22.19 consistent with both single-antigen and dual-antigen approaches.

The clinical outcomes observed in our CART22.19 cohort align with those reported in other dual-targeting CAR T-cell trials.^18 19 21 24^ Initial molecular CR was achieved in 78% of patients, with 12-month EFS and OS rates of 22.2% and 55.6%, respectively. These outcomes are consistent with the bicistronic CD19/CD22 CAR trial by Cordoba *et al* (67% CR, 30% EFS at 12 months). Initial CR rates were also comparable to those reported in the ELIANA study (81%), despite our cohort having received more prior lines of therapy and including patients with CD19-negative disease and/or refractory to tisagenlecleucel. Taken together, these findings highlight the clinical potential of CART22.19 in high-risk pediatric B-ALL populations with otherwise limited treatment options.

Several dual-targeting CAR T-cell strategies engaging CD19 and CD22 have shown limited CD22-mediated functionality, with reduced cytokine release and cytotoxicity on CD22 engagement in bicistronic models, and a predominant dependence on CD19 targeting in cotransduction and coadministration approaches.^17–19^ In contrast, our CART22.19 construct consistently elicited robust T-cell activation and cytokine secretion in response to both CD19^+^ and CD22^+^ targets in vitro. Moreover, the achievement of a complete molecular remission in a patient with CD19-negative disease provides compelling in vivo evidence of effective CD22-mediated antileukemic activity. These findings support the functional competence of the CD22-binding domain in our tandem CAR and highlight its potential to overcome limitations observed in previous approaches.

Despite an initial complete molecular remission rate of 78%, six patients relapsed. In one case, relapse occurred with CD19-negative disease. In contrast, two patients relapsed with CD19^+^CD22^+^ disease and low levels of CART22.19 detectable in peripheral blood, suggesting insufficient functional CAR T-cell persistence as the likely cause of relapse. In patient P9, target antigen expression at the time of relapse was not available; however, relapse occurred after a marked decline in circulating CAR T-cell levels had been observed, further supporting a potential association between loss of CAR T-cell persistence and treatment failure. This pattern is consistent with observations by Ghorashian *et al* and others, in which most relapses occurred despite sustained target antigen expression.^17–19 21^

The mechanisms underlying reduced CAR persistence remain incompletely understood. Our CART22.19 product exhibited a central memory T-cell phenotype in vivo at day 28 postinfusion, a profile previously associated with favorable persistence. However, it may lack the long-term durability of stem-like memory or early central memory T-cell subsets.^31–34^ Additionally, tandem CAR constructs may be susceptible to functional limitations arising from tonic signaling.^20 21^ In our cohort, limited postinfusion monitoring and immunophenotyping restricted comprehensive long-term functional assessment of CART22.19. Although immune-mediated rejection has been proposed in previous studies as a potential cause of limited persistence, this appears less likely, given the use of a fully humanized CAR construct.^35 36^

Notably, Das *et al* recently demonstrated that T-cells collected after multiple cycles of chemotherapy exhibit mitochondrial dysfunction and impaired metabolic reserve, significantly limiting CAR T-cell expansion and persistence.^37^ These observations indicate that the intrinsic quality of autologous T-cells, particularly in heavily pretreated patients, can limit therapeutic efficacy. This finding highlights the multifactorial nature of treatment failure and the need for strategies that enhance T-cell fitness.

One approach to improving efficacy is the use of donor-derived CAR T-cells, leveraging the proliferative and functional advantages of immunocompetent T-cells from healthy donors. The clinical course of patient P1 illustrates the therapeutic benefit of this strategy. After two unsuccessful autoCART22.19 infusions, the patient received RIC alloHSCT in non-remission followed by alloCART22.19 and subsequently achieved long-term remission. In contrast to other allogeneic strategies involving T cell receptor (TCR) editing to prevent GvHD, our strategy preserved the native TCR, minimizing manipulation that may impair CAR T-cell function.^38 39^ Importantly, no unexpected toxicities or GvHD occurred, and durable B-cell aplasia was maintained beyond 4 years after infusion. Consistent with our observations, prior studies in B-ALL and T-cell malignancies have demonstrated that donor-derived CAR T-cell products can induce deep and durable remissions, with superior expansion, persistence, and immune fitness compared to autologous products, particularly in heavily pretreated patients.^40–43^

These findings prompt a reconsideration of treatment sequencing. Prevailing practice favors autologous CAR T-cells followed by consolidative alloHSCT if remission is achieved, in part to mitigate relapse when CAR persistence is limited, with persistence beyond 6 months correlating with long-term survival in BCP-ALL.^44^ However, in our study this sequence did not yield durable benefit, whereas one patient who underwent RIC alloHSCT in non-remission followed by donor-derived CART22.19 achieved a deep and sustained molecular remission with long-term B-cell aplasia. While this observation must be interpreted with caution, it raises the hypothesis that alloHSCT could, in selected cases, serve as a platform enabling donor-derived CAR T-cell therapy with improved persistence and antigen-dependent disease control. Prospective trials that directly compare these sequences are warranted.

Despite these encouraging findings, several limitations must be acknowledged. The named-patient framework introduced heterogeneity in patient characteristics, disease burden, prior therapies, and antigen expression. Case-by-case decision-making, driven by clinical urgency, limited consistency in treatment and follow-up. Furthermore, systematic immunophenotypic characterization of CAR T-cell products, including direct comparisons between autologous and allogeneic products with respect to activation and exhaustion markers, was not feasible in this study and should be addressed in future prospective investigations. The small cohort size and incomplete postrelapse immunophenotyping reduced statistical power and mechanistic insight. Consequently, these results should be interpreted with caution and require validation in prospective, controlled clinical trials.

Building on these findings, we are currently conducting a Phase I trial of a third-generation CD19/CD22 CAR (MB-CART2219.1) in children and adults with relapsed or refractory B-cell malignancies (NCT07108868). The study assesses safety, persistence, pharmacokinetics, and preliminary antitumor activity and evaluates whether engineering strategies to enhance persistence translate into improved long-term outcomes.

## Conclusion

CART22.19 demonstrated a favorable safety profile and promising clinical activity in high-risk pediatric B-ALL. Durable remissions were achieved in a subset of patients, while relapse despite preserved antigen expression indicates limited CAR T-cell persistence as a key challenge. Our findings suggest that donor-derived CAR T-cell therapy following RIC alloHSCT may help address limitations of autologous products, although validation in larger prospective studies is required. Further clinical evaluation of CART22.19 is warranted, with particular emphasis on allogeneic approaches to enhance persistence and improve outcomes in heavily pretreated pediatric leukemia.

## Supplementary material

10.1136/jitc-2025-013901 online supplemental file 1

## Data Availability

Data are available upon reasonable request.
