# Supplementary material for: Human CART22.19 therapy in refractory pediatric B-ALL: insights from a named-patient cohort
Source: J Immunother Cancer. 2026 May 21;14(5):e013901. doi: 10.1136/jitc-2025-013901 (PMC13202116; doi:10.1136/jitc-2025-013901)
Supplement: Supplementary file 1 [file jitc-14-5-s001.docx]

**Supplementary Data**

**Supplementary Methods**

**Design, Vector Production and Preclinical Generation of CAR22.19**

For comparative analyses, a second-generation CAR19 construct was generated alongside the tandem CAR22.19. Both vectors shared an identical lentiviral backbone, with the CAR19 construct containing only the anti-CD19 scFv. CAR sequences were synthesized as gBlocks (Integrated DNA Technologies, Coralville, IA) and cloned into a third-generation lentiviral vector plasmid (Lentigen Technology, Gaithersburg, MD) regulated by the human EF-1α promoter. For the preclinical evaluation, lentiviral particles were produced by transient transfection of HEK 293T cells using a four-plasmid packaging system, as previously described (1). Viral supernatants were harvested, concentrated by centrifugation, and stored at -80 °C until use.

T-cells were isolated from leukapheresis products (HemaCare, Northridge, CA) or buffy coats (Oklahoma City, OK). Human primary CD4^+^ or CD8^+^ cells were isolated from blood products by immunomagnetic separation according to manufacturer’s protocol (Miltenyi Biotec, Bergisch Gladbach), cryopreserved, and later thawed for use. Upon thawing, T-cells were activated using CD3/CD28 MACS GMP T-Cell TransAct reagent and cultured in TexMACS GMP medium (Miltenyi Biotec) supplemented with 30 IU/mL IL-2 at a density of 1 × 10⁶ cells/mL. Lentiviral transduction was performed on day 1 after activation. On day 3, cells were washed and resuspended in fresh IL-2-supplemented TexMACS medium. Cell cultures were maintained at a density of 0.5-2 × 10⁶ cells/mL and functional assessments were conducted between days 8 and 10.

**Flow Cytometric Analysis**

Flow cytometry was used to assess surface molecule expression on CAR T-cells. Cells were stained in cold AutoMACS buffer with 0.5% bovine serum albumin (Miltenyi Biotec), washed, and resuspended in 200 µL Running Buffer (Miltenyi Biotec) for acquisition on a MACSQuant®10 Analyzer. CAR expression was determined using CD22-his (Abcam, Cambridge) or CD19-Fc (R&D Systems, Minneapolis, MN) peptides, followed by secondary staining with anti-His-PE (Miltenyi Biotec) or anti-Fc-AF647 (Jackson ImmunoResearch, West Grove, PA). T-cell subsets were analyzed with anti-CD4 VioBlue (Miltenyi Biotec), and dead cells excluded using Viobility 405/520 Fixable Dye (Miltenyi Biotec).

***In Vitro* Cytotoxicity and Cytokine Secretion** **Assays**

Cytotoxicity assays were performed to evaluate the functional activity of CAR T-cell constructs. Firefly luciferase–expressing target cells and the corresponding knockout cell lines were generated and validated as previously described (2). CAR T-cells were co-cultured for 16 hours with 5,000 target cells stably expressing firefly luciferase at the indicated E:T ratios. Following incubation, Steady-Glo® reagent (Promega, Madison, WI) was added to each well, and luminescence was measured using a GloMax Luminometer (Promega) and reported as counts per second (CPS). Maximum and minimum signals were established using target-only wells treated with or without 1% Tween-20, respectively. Specific lysis was calculated using the formula: % specific lysis = 1 - (sample CPS - min CPS) / (max CPS - min CPS).

Supernatants collected from co-cultures were analyzed for cytokine release using ELISA kits (Thermo Fisher Scientific, Waltham, MA) to quantify IL-2 and IFN-γ concentrations.

**NSG Mice Experiments**

All animal experiments were conducted at MI Bioresearch (now part of LabCorp; Ann Arbor, MI) in accordance with protocols approved by the Institutional Animal Care and Use Committee (IACUC) and in full compliance with applicable laws, regulations, and National Institutes of Health (NIH) guidelines. Female NSG mice (NOD.Cg-Prkdc^scid^ Il2rg^tm1Wjl^/SzJ; n=22), aged 6–7 weeks, were intravenously injected with 1 × 10⁶ NALM-6 cells, a model of B-cell precursor leukemia. Tumor burden was monitored via BLI using the Xenogen IVIS-200 system (PerkinElmer, Shelton, CT). On day 3 post-injection, mice with comparable baseline tumor burdens were randomized to tumor-only (n=6), untransduced T cells [UTD] (n=6), CAR19 (n=4), or CART22.19 (n=6); group sizes were chosen for exploratory purposes and no a priori sample-size calculation was performed. Treatment groups received 1 × 10⁷ CAR T-cells per mouse. Tumor progression was monitored at prespecified intervals (days 0, 3, 7, 14, 21, 28, and 37) using *in vivo* imaging. Peripheral blood was collected on day 28 after CAR T-cell administration and analyzed by flow cytometry. Mice were euthanized upon reaching humane endpoints (excessive tumor burden or clinical morbidity) or on day 31 after NALM-6 infusion, whichever occurred first; animals were monitored daily and imaged at the indicated time points. Statistical significance was evaluated with a mixed-effects model using the Geisser-Greenhouse correction. No mice or data points were excluded from analysis. Potential confounders were not controlled, and groups were not blinded.

**Quality Control of CART22.19 Products**

Before infusion, clinical CART22.19 products underwent standard quality control evaluations, including sterility testing, assessment of cell viability, and measurement of transduction efficiency. Microbiological testing was conducted in accordance with GMP guidelines. Mean cell viability at the time of product release was 99.7% (range, 99.4-99.8%). Transduction efficiency was evaluated by flow cytometry using CD19-Fc and CD22-his detection reagents, with a mean transduction rate of 56.7% (range, 48.3-70.8%) at harvest. A summary of in-process controls and final product testing is provided in **Supplementary Table S1**.

**Immunophenotyping of Leukemic Blasts**

Bone marrow aspirate samples obtained at initial diagnosis and relapse were analyzed by flow cytometry at accredited laboratories of the University Hospital Frankfurt, following the protocol described by Karawajew et al (3). Leukemic blasts were identified based on the expression profile of CD19, CD22, CD24, CD25, CD10, CD20, CD34, CD38, and CD44. Antigen expression intensity was evaluated in accordance with the guidelines established by Dworzak et al (4).

**Immune Monitoring**

Immune monitoring of CART22.19 was performed by flow cytometric analysis of peripheral blood samples collected at multiple time points following infusion. Red blood cells were lysed prior to staining (Red Blood Cell Lysis Solution, Miltenyi Biotec). Subsequently, 100 µL of lysed whole blood was incubated with 2 µL of either CD19-CAR or CD22-CAR detection reagent (Miltenyi Biotec) for 10 minutes at room temperature. Following two washes with CliniMACS PBS/EDTA buffer (Miltenyi Biotec), cells were stained with a fluorochrome-conjugated antibody panel comprising anti-Biotin-PE (Miltenyi Biotec), CD45-VioBlue (Miltenyi Biotec), CD3-FITC (Miltenyi Biotec), CD4-VioGreen (Miltenyi Biotec) and CD8-APC-Vio770 (Miltenyi Biotec) followed by an additional wash in CliniMACS PBS/EDTA. 7-AAD viability dye (Miltenyi Biotec) was added 5 minutes prior to sample acquisition.

Samples were acquired on a MACSQuant® flow cytometer (Miltenyi Biotec), and data were analyzed using FlowJo software version 10.8 (BD, Ashland, OR). The gating strategy is provided in **Supplementary Figure S4D**.

**Therapy Response Assessment**

Therapeutic response was evaluated through serial bone marrow aspirates and, in patients with a history of CNS involvement, cerebrospinal fluid analysis. MRD in the bone marrow was assessed by real-time quantitative PCR when patient-specific molecular markers were available, or by multiparameter flow cytometry otherwise. MRD evaluations were performed at accredited laboratories at University Hospital Frankfurt, with flow cytometric analysis conducted according to the protocol described by Karawajew et al (3). MRD negativity was defined as <10⁻⁴ leukemic cells by PCR or <0.01% blasts by flow cytometry. In cases of suspected or confirmed extramedullary involvement, imaging studies (including MRI, CT, PET-MRI, or PET-CT) were performed every three months or as clinically indicated. Complete remission was defined as the absence of detectable extramedullary disease and MRD negativity in the bone marrow.

CART22.19 persistence in peripheral blood was assessed by flow cytometry at variable intervals, as described in the Immune Monitoring section. Detection of CAR T-cells was defined as ≥0.01% of CD3^+^ cells. CART22.19 measurements were performed for a median duration of 70 days (range: 14-176 days) following infusion. In parallel, peripheral blood flow cytometry for CD45, CD3, and CD19 was used to quantify circulating B-cells as a surrogate marker of CAR T-cell activity. Longitudinal kinetics of CAR T-cell persistence are shown in **Figure 2E**.

**Safety Monitoring and Adverse Event Assessment**

CRS and ICANS were graded according to the 2019 consensus criteria of the ASTCT (5). All other AEs and clinically significant laboratory abnormalities were evaluated using the CTCAE version 5.0 (6).

Laboratory safety monitoring was conducted on blood samples obtained prior to, during, and following CART22.19 infusion. Routine assessments included blood counts, coagulation profiles, renal and hepatic function panels and inflammatory markers such as IL-2 and IL-6, measured every other day for a minimum of one month. Additional laboratory evaluations, including microbiological and infectious disease testing, were performed twice weekly or as clinically indicated throughout the post-infusion period.

Management of grade ≥3 CRS included intravenous dexamethasone, tocilizumab and/or anakinra. As seizure prophylaxis, some patients were preemptively treated with levetiracetam. Anemia and thrombocytopenia were managed with red blood cell and platelet transfusions, respectively.

**Patient P1: Reduced-Intensity Conditioning, alloHSCT Graft Composition, and CAR T Cell Manufacturing**

The RIC regimen for patient P1 included fludarabine (120 mg/m² BSA), melphalan (140 mg/m² BSA), and anti-thymocyte globulin (ATG; 30 mg/kg BW), followed by alloHSCT from a 9/10 HLA-matched, ABO-incompatible, CMV-seropositive unrelated donor (recipient: O Rh⁺; donor: O Rh⁻).

HLA typing of patient P1 showed A*01:01, *68:02; B*41:01, *18:01; C*17:01, *07:04; DRB*107:01, *03:01; and DQB*103:03, *02:01. The donor was typed as A*01:01, *68:02; B*18:01, *41:01; C*05:01, *17:01; DRB*103:01, *07:01; and DQB*102:01, *03:03. Comparison of recipient and donor HLA typing revealed a single mismatch at HLA-C.

The graft was derived from donor PBSCs and contained 30.2% CD3⁺, 7.2% CD19⁺, 0.4% CD34⁺, 3.1% CD56⁺CD3⁻, 34.8% CD14⁺, 29.5% TCRαβ⁺, and 0.7% TCRγδ⁺. After TCRαβ depletion, the graft composition was 1% CD3⁺, 0.003% CD19⁺, 0.73% CD34⁺, 3.55% CD56⁺CD3⁻, 57.7% CD14⁺, 0.0006% TCRαβ⁺, and 1% TCRγδ⁺.

The patient received a total of 600 × 10⁶ cells/kg BW (3.24 × 10¹⁰ cells), including CD34⁺ 4.38 × 10⁶/kg (2.37 × 10⁸ cells), CD3⁺ 5981 × 10³/kg (3.23 × 10⁸ cells), CD56⁺ 21.44 × 10⁶/kg (1.16 × 10⁹ cells), and TCRαβ⁺ 3.74 × 10³/kg (2.02 × 10⁵ cells).

Granulopoiesis was stimulated with G-CSF from day +4 after alloHSCT to day +11. Granulocyte engraftment occurred on day +10, and leucocyte engraftment on day +11. At the time of alloCART22.19 infusion, 72 days post-alloHSCT, the patient presented with 1,690 leukocytes/μL, including 114 CD3⁺/μL, 13 CD4⁺/μL, 41 CD8⁺/μL, and 239 CD56⁺CD3⁻/μL.

For alloCART22.19 manufacturing, cells were derived from the same donor apheresis used for graft preparation. A fraction of the material was cryopreserved on the day of TCRαβ depletion and subsequently thawed for CAR T-cell generation. The starting product comprised 1.041 × 10⁹ CD3⁺ cells, 246.4 × 10⁶ CD19⁺ cells, and 0.1 × 10⁹ CD56⁺CD3⁻ cells. Written informed consent for CAR T-cell production was obtained from the donor.

**Supplementary Tables and Figures**

***Supplementary Table S1: Manufacturing Parameters and Product Characteristics of Patient-Specific CART22.19***

| Patient | Recovery after enrichment [%] | Day 5 cell count [x10^6^] | Day 5 transd. efficiency | Harvest day | Harvest cell count [x10^8^] | Harvest transd. frequency [%] | Vitality frequency [%] |
| --- | --- | --- | --- | --- | --- | --- | --- |
| P1 | 27.2 | 122 | 48.4 | 9 | 11.5 | 49 | 99.8 |
| P2 | 43.6 | 290 | 52 | 11 | 19.3 | 48.3 | 99.4 |
| P3 | 68.7 | 290 | 61.5 | 11 | 21.4 | 59.2 | 99.8 |
| P4 | 34 | 165 | 43.4 | 11 | 19.7 | 49.2 | 99.6 |
| P5 | 69.9 | 270 | 46.4 | 7 | 8.2 | 49.3 | 99.7 |
| P6 | 58.7 | 282 | 60.1 | 12 | 33.5 | 69 | 99.8 |
| P7 | 62.5 | 492 | 74 | 12 | 37.4 | 70.8 | 99.7 |
| P8 | 64.7 | 455 | 53 | 12 | 26.6 | 53.1 | 99.8 |
| P9 | 63.3 | 306 | 57.8 | 12 | 25.6 | 62 | 99.4 |

Abbreviations: transd: transduction.

***Supplementary Table S2: Detailed Clinical Characteristics and Outcomes of Pediatric Patients Treated with CART22.19.***

| Patient ID | P1 | P2 | P3 | P4 | P5 | P6 | P7 | P8 | P9 |
| --- | --- | --- | --- | --- | --- | --- | --- | --- | --- |
| Sex | F | M | M | M | F | M | M | M | F |
| ALL subtype | pre-B ALL | c-ALL | pre-B ALL | BCP ALL | c-ALL | pre-B ALL | c-ALL | c-ALL | c-ALL |
| Specific genetic alterations | Het FANCM variants us | KMT2A rear-range-ment | None | N/A | t(9;22) BCR-ABL1 | CDKN-2A/B deletion | t(1;19) PBX1 | IZKF1 deletion, SPINK5 mutation | ETV6-RUNX1 |
| Age at diagnosis [years] | 16.6 | 0.2 | 3.5 | N/A | 2.3 | 13 | 2.1 | 10.7 | 15.1 |
| Prior lines of therapy | 5 | 1 | 4 | 7 | 6 | 6 | 3 | 2 | 2 |
| Prior anti CD19/22 therapy | Yes (Blina/  Ino) | No | Yes (Blina/  Ino/  CAR19) | Yes (CAR19) | Yes (Blina/  Ino) | Yes (Blina/  Ino/  CAR19) | No | Yes (Blina) | Yes (Blina/  Ino) |
| Nr. of prior relapses | 2 | 1 | 4 | 6 | 4 | 3 | 3 | 0 | 1 |
| Prior HSCT | 0 | MUD | MUD | MUD/ MMFD | MSD | MMFD | 2x MSD | 0 | 0 |
| Indication for CART22.19 | CD19 neg blast pop | CD19 neg blast pop | Relapse after CAR19, CD19^-^ blast pop | Relapse after CAR19 | No access to CAR19 | Relapse after CAR19, CD19^-^ blast pop | No access to CAR19 | No access to CAR19 | CD19 neg blast pop |
| Initial diagnosis to CART22.19 [months] | 24 | 13 | 71 | N/A | 58 | 57 | 72 | 8 | 46 |
| CART22.19 source | 2x Auto / 1x MUD | Auto | Auto | Auto | MSD | MMFD | Auto | 3x Auto | Auto |
| Status before CART22.19 | MRD 2*10^-3^ / MRD 5*10^-3^ / MRD 2*10^-3^ | 60% blasts in BM | 3% blasts in BM | MRD 2.5*10^-1^ | MRD^-^ | 64% blasts in PB | 89% blasts in BM | MRD 10^-2^ / MRD 2*10^-3^ / MRD 10^-5^ | 69% blasts in PB |
| EMD / CNS disease before CAR22.19T [yes=1/ no=0] | 1 / 0 | 1 / 0 | 0 / 1 | 0 / 0 | 0 / 1 | 0 / 0 | 0 / 0 | 0 / 0 | 0 / 0 |
| Dose level [x10^6^/kg BW] | 3 / 6 / 3 | 3 | 3 | 3 | 1 | 1 | 3 | 3 / 0.3 / 5 | 3 |
| Initial response to CART22.19 | CR / PD / CR | CR | PD | CR | PD | CR | CR | CR / PD / CR | CR |
| Relapse after CART22.19 [yes=1/ no=0] | 0 / 0 / 0 | 0 | 0 | 1 | 0 | 1 | 1 | 0 / 0 / 0 | 1 |
| Time to relapse [months] | -- | -- | -- | 2 | -- | 10 | 10 | 4 | 10 |
| CRS [grade] | 3 / 0 / 3 | 1 | 0 | 2 | 2 | 3 | 1 | 4 / 2 / 0 | 4 |
| ICANS [grade] | 0 / 0 / 0 | 0 | 0 | 1 | 4 | 0 | 0 | 0 / 0 / 0 | 1 |
| Further HSCT | MUD | MMFD | -- | -- | -- | HSCT | MMFD | MMFD | MMFD |
| Deceased/ alive [1/0] | 0 | 0 | 1 | 1 | 1 | 1 | 0 | 0 | 0 |

Abbreviations: Allo: allogeneic; ALL: acute lymphoblastic leukemia; Auto: autologous; BCP: B-cell precursor; Blina: blinatumomab; BM: Bone marrow; BW: body weight; c-ALL: common acute lymphoblastic leukemia; CAR19: CD19 directed CAR T-cells; CNS: Central nervous system; CR: complete remission; CRS: cytokine release syndrome; EMD: Extramedullary disease; F: female; Het: heterozygous; HSCT: hematopoietic stem cell transplantation; ICANS: immune effector cell-associated neurotoxicity syndrome; Ino: inotuzumab ozogamicin; M: male; MMFD: mismatched family donor; MRD^-^: minimal residual disease negative; MSD: matched sibling donor; MUD: matched unrelated donor; neg: negative; N/A: information not available; NR: no remission; PD: persistent/progressive disease; PB: Peripheral blood; pop: population; pre-B ALL: precursor B acute lymphoblastic leukemia; Us: uncertain significance.

**Supplementary Figure S1**

**A-B** CART22.19, CAR19, or UTD were co-cultured with target cell lines at indicated E:T ratios for 16 hours. Supernatants were collected and analyzed by ELISA to quantify IL-2 (**A**) and IFN-γ (**B**) secretion. Target cell lines included Raji WT (CD19⁺CD22⁺), Raji CD19^KO^ (CD19⁻CD22⁺), Raji CD22^KO^ (CD19⁺CD22⁻), and Raji CD19^KO^CD22^KO^ (CD19⁻CD22⁻). Data represent mean ± SD of three technical replicates. Statistical significance was determined using unpaired t-tests. **C-D** On day +28, peripheral blood from NSG mice was collected for flow cytometric analysis. **C** Total number of CAR^+^ T-cells in 50 µL of peripheral blood. Each data point represents an individual mouse (n=4-6 per group); data shown as mean ± SEM. **D** Total number CAR^+^ central memory T-cells (CD62L⁺CD45RO⁺) in 50 µL of peripheral blood. Each data point represents an individual mouse (n=4-6 per group); data shown as mean ± SEM. Statistical comparisons were performed using unpaired student’s t-tests. Abbreviations: CAR: chimeric antigen receptor; CAR19: CD19-directed CAR; CART22.19: fully human tandem CAR T-cells with anti-CD22 and anti-CD19 scFvs; Conc.: concentration; ELISA: enzyme-linked immunosorbent assay; E:T: effector-to-target ratio; IFN-γ: interferon gamma; IL-2: interleukin-2; KO: knockout; ns: not significant; NSG: NOD scid gamma; SD: standard deviation; SEM: standard error of the mean; UTD: untransduced activated T-cells; WT: wild type.

**Supplementary Figure S2**

Representative flow cytometry gating strategy exemplified for patient P9, as applied to all patients to assess CD19 and CD22 expression on leukemic blasts in bone marrow. **A** Leukocytes were first gated based on FSC-H vs. SSC-H, and blasts were identified as a CD45^dim^ population expressing CD10, followed by evaluation of CD19 expression. **B** In a parallel analysis, leukocytes were gated as above, and blasts were defined as CD34^weak^. Co-expression of CD19 and CD22 was then assessed. Sequential gating steps are indicated by red arrows. Abbreviations: FSC-H: forward scatter height; SSC-H: side scatter height.

**Supplementary Figure S3**

Subgroup analysis of patients with ongoing response following CART22.19 therapy. Forest plot illustrating the proportion of patients with ongoing remission (P1, P2) across clinical and treatment-related subgroups. Stratification variables included age, disease classification, prior lines of therapy, number of previous relapses, history of allogeneic stem cell transplantation, indication for CAR T-cell therapy, interval from initial diagnosis to CAR T-cell infusion, and CAR T-cell dose level. Data are presented as percentages with 95% CI calculated using the Clopper-Pearson method. Each dot represents the mean proportion of patients with sustained response within the respective subgroup. Abbreviations: ALL: acute lymphoblastic leukemia; BW: body weight; CAR: chimeric antigen receptor; CART22.19: fully human tandem CAR T-cells with anti-CD22 and anti-CD19 scFvs; CI: confidence interval; HSCT: hematopoietic stem cell transplantation.

**Supplementary Figure S4**

Detailed clinical and immunological course of patient P1 is shown, including longitudinal monitoring of disease status, cytokine release, CAR T-cell dynamics, and representative flow cytometry gating strategy. **A** MRD levels in bone marrow, assessed by RT-PCR at the indicated time points, referenced to months since first CART22.19 administration. Colored dots correspond to the timing of therapeutic interventions summarized in **Figure 3A**. **B** Plasma cytokine levels post-infusion of CART22.19. Concentrations of IL-6 and sIL-2R were measured in peripheral blood plasma at the indicated time points. **C** Longitudinal monitoring of CART22.19 in peripheral blood. PBMCs were analyzed by flow cytometry post-infusion. The absolute number of viable CART22.19⁺ cells per µL blood was determined using a CD19-scFv detection reagent. Data are shown for total T-cells (CD3⁺, red), cytotoxic T-cells (CD3⁺CD8⁺, dark red), and T helper cells (CD3⁺CD4⁺, yellow). **D** Representative flow cytometry gating strategy used to assess CART22.19 in peripheral blood. Abbreviations: 7AAD: 7-aminoactinomycin D; CAR: chimeric antigen receptor; CART22.19: fully human tandem CAR T-cells targeting CD19 and CD22; FSC: forward scatter; IL-6: interleukin-6; MRD: minimal residual disease; MUD: matched unrelated donor; PBMC: peripheral blood mononuclear cell; RT-PCR: reverse transcriptase polymerase chain reaction; scFv: single-chain variable fragment; sIL-2R: soluble interleukin-2 receptor; SSC: side scatter.

**Supplementary Figure S1**

**Supplementary Figure S2**

**Supplementary Figure S3**

**Supplementary Figure S4**

**References**

1.	Merten OW, Hebben M, Bovolenta C. Production of lentiviral vectors. Mol Ther Methods Clin Dev. 2016;3:16017.

2.	Atar D, Mast AS, Scheuermann S, Ruoff L, Seitz CM, Schlegel P. Adapter CAR T Cell Therapy for the Treatment of B-Lineage Lymphomas. Biomedicines. 2022;10(10).

3.	Karawajew L, Dworzak M, Ratei R, Rhein P, Gaipa G, Buldini B, et al. Minimal residual disease analysis by eight-color flow cytometry in relapsed childhood acute lymphoblastic leukemia. Haematologica. 2015;100(7):935-44.

4.	Dworzak MN, Buldini B, Gaipa G, Ratei R, Hrusak O, Luria D, et al. AIEOP-BFM consensus guidelines 2016 for flow cytometric immunophenotyping of Pediatric acute lymphoblastic leukemia. Cytometry B Clin Cytom. 2018;94(1):82-93.

5.	Lee DW, Santomasso BD, Locke FL, Ghobadi A, Turtle CJ, Brudno JN, et al. ASTCT Consensus Grading for Cytokine Release Syndrome and Neurologic Toxicity Associated with Immune Effector Cells. Biol Blood Marrow Transplant. 2019;25(4):625-38.

6.	Services USDoHaH. Common Terminology Criteria for Adverse Events (CTCAE) Version 5.0: National Cancer Institute; 2017 [Available from: <https://ctep.cancer.gov/protocoldevelopment/electronic_applications/ctc.htm>.
